# Treatment of Poisoning by Chloral Hydrate

**Published:** 1894-01-20

**Authors:** Benjamin Ward Richardson


### Treatment of Poisoning by Chloral Hydrate.

By Sir BENJAMIN WARD RICHARDSON, M.D., F.R.S.

I TAKE to myself some blame for allowing other subjects to occupy me so much as to prevent me republishing, in a more accessible form, the rules I laid before the British Association in 1871 on the treatment of patients accidentally poisoned by chloral hydrate. The treatment, which is described in the Transactions of the Association for 1871, p. 145-47, is detailed with much care; it was founded on experimental data: and as it has, since that time, been put to the practical test in three instances with entire success, it may be useful to describe it here. I found that chloral hydrate, owing to its great solubility, is so rapidly absorbed and diffused in and through the tissues of living animals, that it is vain to attempt its removal by the ordinary methods of removing a poison, however it may have been introduced. The animal or person under the influence of chloral, like an animal or person in a fever, must go through a series of stages on the way to recovery or death; and these stages will be long or short, slightly dangerous or intensely dangerous, all but fatal or actually fatal, according to the conditions in which the body subjected to chloral is surrounded.

I found that one of the first and most marked symptoms or effects of chloral is reduction of the animal temperature, and that when an animal is deeply under the influence of the narcotic, the temperature of its body, unless the external warmth be carefully sustained, will quickly descend seven and even eight degrees Fahrenheit below the natural standard. This fall of temperature was sure to lead to two most serious consequences: it allowed condensation of fluid on the bronchial pulmonary surface, *hydrops bronchialis*, with the attendant asphyxia from exclusion of air into the vesicular structure, and it induced the convulsion of cold, a convulsion which sharply precedes death.

The experiments illustrating these effects were very precise in their meaning, I found that in animals that have normally the temperature of their body very high, pigeons for example—109 degrees Fahrenheit—the death or the recovery from a large dose of chloral could be determined by the degree of heat to which they were exposed. Left at a low degree, 40 degrees Fahrenheit, death would follow in one; while another receiving the same dose at the same time, if exposed to a temperature of 95 degrees Fahrenheit, would certainly recover.

I discovered also that in an animal under the profound action of chloral, the process of waste, indicated by loss of weight although less than is common under the usual condition of life, was still considerable; but that under the sleep the digestive and assimilative powers were but slightly impaired. Hence, if two similar animals were cast into a deep sleep by an excessive dose of the narcotic, and the one was left without food while the other was systematically fed by artificial means, one-fourth of additional chance of recovery would be given to the animal that was supplied with such food.

From these observations I was led to the following conclusions as to treatment in cases of chloral poisoning in the human subject:—

1. Avoid all disturbance of the stomach by attempts to evacuate it; and specially avoid emetic remedies, since nothing depresses so much and assists in reducing animal temperature as vomiting.

2. Place the body at once in a warm atmosphere; keep the body itself very warm, but of all things see that a warm atmosphere is inspired. The air to be breathed should be raised and sustained at 90 degs. Fahrenheit at least.

3. Be most careful in sustaining the body by the administration of warm food. Hot milk, to which a little lime water has been added, is the best food that can be supplied. If it is impossible to make the patient swallow, the milk is easily introduced into the stomach by the long nostril tube. It need not be thrown in rashly, but may be made to enter in a gentle current. It is good practice also to administer frequently an enema of beef tea or of milk and water as warm as it can be borne, the heat being of as great value as the food that may be absorbed by the bowel.

4. A fourth point to remember is to sustain the breathing should that begin to fail. I found invariably in the lower animals under the profound influence of choral, that the respiration always ceased before the motion of the heart. This was so decidedly the case that in some instances, in which every sign of life had passed away, it was observed, on laying open the chest for post-mortem purposes, that the muscular walls of the cardiac cavities still showed signs of irritability. Hence the importance of gentle artificial respiration. No complicated artificial apparatus is demanded. A pair of small parlour bellows suffices. To the nozzle of such bellows attach an indiarubber tube, and at the other or free end a nostril tube. Place the nostril tube in one nostril, close the other nostril by pressure, close the mouth, and by the slow action of the bellows gently inflate the chest; then, remove the nostril tube for the moment, allow the chest to empty itself, and repeat. One charge of air, given at intervals of ten seconds from each other, and without the least violence, is all sufficient, since a small quantity of air is necessary to keep up the respiration as would be required if the body were in a state of catalepsy or of hybernation.

These are the practical means to be employed. Antidotes there are none, nor can we form any reasonable idea of an antidote. Recovery from chloral is a systematic process. The chloral has to be decomposed by the alkaline blood, the chloroform that is liberated in the decomposition has to be eliminated from the body by the lungs and the skin, and the formate of soda that is formed has to escape by the skin, bowels, and kidney. In proportion as these eliminations take place, under warmth and sustainment of food, safety is insured. I calculated that an adult person who has taken sufficient chloral to be narcotized by it should be able to dispose of it at the rate of seven grains per hour if favourably placed for elimination.

One detail more. It is very important all through the process to see that the bladder is kept free from distension, and to relieve it, from time to time, by the catheter, if there be no natural micturition.

